# Design and Engineering of “Green” Nanoemulsions for Enhanced Topical Delivery of Bakuchiol Achieved in a Sustainable Manner: A Novel Eco-Friendly Approach to Bioretinol

**DOI:** 10.3390/ijms221810091

**Published:** 2021-09-18

**Authors:** Agnieszka Lewińska, Marta Domżał-Kędzia, Ewa Maciejczyk, Marcin Łukaszewicz, Urszula Bazylińska

**Affiliations:** 1 Faculty of Chemistry, University of Wroclaw, Joliot-Curie 14, 50-383 Wroclaw, Poland; 2 Department of Biotransformation, Faculty of Biotechnology, University of Wroclaw, Joliot-Curie 14a, 50-383 Wroclaw, Poland; marta.domzal@uwr.edu.pl (M.D.-K.); marcin.lukaszewicz@uwr.edu.pl (M.Ł.); 3 Institute of Natural Products and Cosmetics, Faculty of Biotechnology and Food Sciences, Lodz University of Technology, Stefanowskiego 2/22, 90-924 Lodz, Poland; ewa.maciejczyk@p.lodz.pl; 4 Laboratory of Nanocolloids and Disperse Systems, Department of Physical and Quantum Chemistry, Faculty of Chemistry, Wroclaw University of Science and Technology, Wybrzeze Wyspianskiego 27, 50-370 Wroclaw, Poland

**Keywords:** local delivery, nanoformulations, nanomedicine, supercritical CO_2_ extraction, surfactin, coco-betaine, biosurfacants skin application, HaCaT cell line, NHDF cell line

## Abstract

In the present work, we establish novel “environmentally-friendly” oil-in-water nanoemulsions to enhance the transdermal delivery of bakuchiol, the so-called “bioretinol” obtained from powdered *Psoralea corylifolia* seeds via a sustainable process, i.e., using a supercritical fluid extraction approach with pure carbon dioxide (SC-CO_2_). According to Green Chemistry principles, five novel formulations were stabilized by “green” hybrid ionic surfactants such as coco-betaine—surfactin molecules obtained from coconut and fermented rapeseed meal. Preliminary optimization studies involving three dispersion stability tests, i.e., centrifugation, heating, and cooling cycles, indicated the most promising candidates for further physicochemical analysis. Finally, nanoemulsion colloidal characterization provided by scattering (dynamic and electrophoretic light scattering as well as backscattering), microscopic (transmission electron and confocal laser scanning microscopy), and spectroscopic (UV–Vis spectroscopy) methods revealed the most stable nanocarrier for transdermal biological investigation. In vitro, topical experiments provided on human skin cell line HaCaT keratinocytes and normal dermal NHDF fibroblasts indicated high cell viability upon treatment of the tested formulation with a final 0.02–0.2 mg/mL bakuchiol concentration. This excellent biocompatibility was confirmed by ex vivo and in vivo tests on animal and human skin tissue. The improved permeability and antiaging potential of the bakuchiol-encapsulated rich extract were observed, indicating that the obtained ecological nanoemulsions are competitive with commercial retinol formulations.

## 1. Introduction

Scientific investigations on sustainable processes in nanoemulsion formulations as kinetically stable oil-water dispersions with droplet size 20–500 nm [1,2,3], and progress in technologies for eco-friendly and non-toxic materials became the driving forces for the growing interest in using surfactants with a more “green” nature [4,5]. Recently, this group of “greener” semi-synthetic materials has experienced significant development in Europe and North America as a result of increasing applications in personal care, pharmaceutics, and agrochemicals. Consequently, the chemical industry is now forced to implement strategies for producing more sustainable products through more environmentally friendly processes without sacrificing product performance [6].

There are at least two strategies for replacing synthetic surface-active agents. Firstly, bio-based products can be introduced into the surfactant molecule, e.g., lauryl acid derivatives, which attenuate the synthetic nature, toxicity, and hazardousness of traditional chemical technologies [6,7]. A good example is the manufacture of semi-synthetic coco betaine—a very mild amphoteric surfactant derived from coconut oil and dimethylaminopropylamine. This “vegan” friendly product, classified as amphoteric or zwitterionic surfactant, has low skin and ocular irritation properties and is not affected by water hardness, producing excellent foam with good stability in soft and hard water [7,8]. The second approach consists of the application of completely natural bio-based products, involving sustainable fabrication of biosurfactant-secondary metabolites produced at the end of the exponential growth phase of microorganisms, as a more necessary biotechnological alternative [5,6,9]. Biosurfactants, also known as microbial surfactants, are generally considered less toxic and more biodegradable, exhibiting similar functionalities to synthetic analogues, while they can be obtained from renewable sources. Also, they show better environmental compatibility, higher foaming, and usually remain stable at wide ranges of pH and temperatures. Biosurfactants may also exhibit high surface activity at low critical micellar concentrations (CMCs) by spontaneously adsorbing at various interfaces, and they self-assemble in an aqueous solution [6,9,10]. Thus, they may protect nanoemulsion physical stability and prevent deterioration of entrapped bioactive molecules during processing and storage.

In particular, surfactin, a highly surface-active cyclic lipoheptapeptide produced by *Bacillus subtilis*, is a fascinating biomolecule able to reduce the surface tension of water from 72 to 27 mN/m at concentrations as low as 10 mg/L. Moreover, surfactin exhibits antibacterial, antifungal, antiviral, and even antitumor activities, while demonstrating a supporting role in collagen production [10,11,12]. This unique feature, combined with excellent emulsification properties and its difficult-to-break traditional emulsion, may thus be beneficial in surfactin, assisting in the nanoemulsion stabilization process of other surface-active compounds; for example, enhancing the moderate emulsifying properties of coco-betaine, and the application of these green formulations in skin care products, for potential delivery of bioactive compounds. In fact, both surfactants are ionic, which is necessary for obtaining highly stable formulations, since the stability of any nanoemulsion is more complex when electrostatic effects are present [13]. Thus, using these hybrid-surface actives for new “green” nanosystem stabilization could be likewise advantageous.

**Scheme 1:**
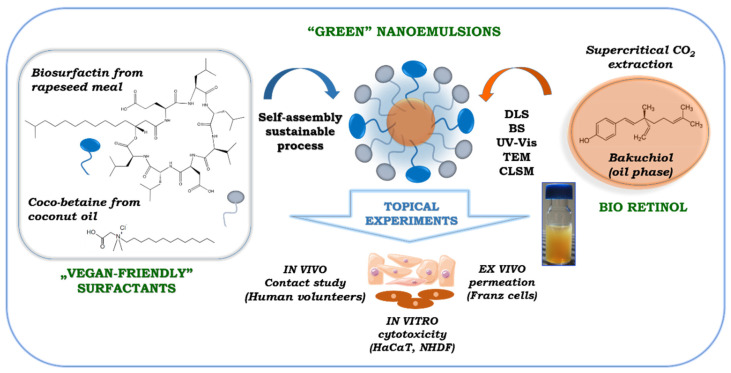
A general outline of the performed studies.

With this in mind, we have designed and engineered a novel oil-in-water nanoemulsion platform (Scheme 1) stabilized by two surface-active agents with a “green” nature, i.e., ecological semi-synthetic coco-betaine and bioinspired rapeseed-origin surfactin. The proposed formulation has a high potential to significantly boost the solubility and action effectiveness of poorly water-soluble compounds, due to its increased surface-to-volume ratio, smaller particle size, and greater mobility, as well as enhanced stability and protection against premature degradation—the key parameters characteristic of nanoemulsion systems [2,3]. Moreover, the proposed nanoemulsions were fabricated based on recent trends for accessing sustainable materials from renewable sources (surfactants, bioactives), maintaining sustainable development, and Green Chemistry procedures, mostly due to the involvement of a low-energy self-assembly approach. Thus, water surfactin–coco-betaine nanosystems were developed for efficient transdermal delivery of bakuchiol—a vegetal functional compound with anti-oxidant, anti-inflammatory, anti-bacterial, and anti-cancer activity.

Bakuchiol is the natural alternative to vitamin A, and thus is so-called “bioretinol”, since it helps to provide the same benefits but with less harmful side effects [14,15]. It is a terpene phenol isolated in 1966 from bakuchi seeds (*Psoralea corylifolia*, L.), and belongs to an important group of compounds in the class of natural products referred to as meroterpenoids. *P. corylifolia* is widely used in Indian (Ayurvedic) and Chinese medicine to treat many diseases. The seeds have been used to treat psoriasis, leucoderma, leprosy, acne, and inflammatory diseases of the skin, and as an aphrodisiac, anthelmintic, laxative, and diuretic. Pure bakuchiol has been reported to protect squalene and many skin lipids from oxidation due to its excellent antioxidant activity. Furthermore, it possesses anti-inflammatory, anti-bacterial, anti-tumor, hepatoprotective, and caspase-3-dependent apoptosis activity. The cytotoxicity of bakuchiol relates to its DNA polymerase 1 inhibition [16,17].

The specific novelty of our studies involves bakuchiol extraction by supercritical fluid, using carbon dioxide (SC-CO_2_) as a “green” sustainable alternative to traditional solvent-based extraction methods. Supercritical fluid properties are intermediate between typical gases and liquids, which could be more advantageous than conventional approaches. Extraction of SC-CO_2_ operates at low temperatures and short extraction times; thus, the thermal and oxygen degradation of bioactive compounds can be minimized [18,19]. Furthermore, the process involves using CO_2_ as a non-toxic, non-flammable, odorless, tasteless, inert, inexpensive solvent with convenient critical properties and good solubility of hydrophobic compounds contained in various plants [18,19,20]. The main advantages of SC-CO_2_ lay in the higher mass transfer rates, increased selectivity, and lower organic solvent consumption. Thus, this “green” method of separation has gained remarkable interest among the scientific community in recent years for producing other biological products [21,22]. So far, a few research teams have investigated the concept of supercritical fluid extraction (SFE) of furocoumarins from plant materials. However, they only focused on obtaining the extracts, their characterization, and the selection process parameters without any implementation in nanoemulsion or other delivery system formulations [23,24].

The recommended sustainable products formulated via a sustainable self-assembly approach exhibit appropriate size, good physical stability, biocompatibility with human skin cell lines (HaCaT keratinocytes and NHDF normal human dermal fibroblasts), and improved skin permeability of the encapsulated cargo, and thus prove themselves as interesting environmentally friendly candidates for preserving the unique biological activity of vegetal retinol. To the best of our knowledge, the presented formulations are the first example of “vegan-friendly” nanoemulsions with sustainably achieved bakuchiol stabilized by hybrid coco-betaine–surfactin molecules dedicated to topical applications.

## 2. Results and Discussion

It has been found that skin, as the largest organ of the human body, is the most popular route of non-invasive administration of many bioactive compounds. Topical delivery is a promising alternative to oral, intravenous, and parenteral distribution due to its low invasiveness and painfulness [1]. Nevertheless, most of the active molecules face challenges in low penetration across the *stratum corneum* (SC)—the outer skin layer—limiting their permeation, causing a huge problem in this route of administration [8]. The newest investigations indicate nanoemulsions as effective “soft” lipid formulations for improving penetration of drugs and other active agents via the SC and into deeper layers of skin, leading to prolonged cargo release in transdermal delivery [1,25]. Thus, in the present contribution, we have designed and engineered a novel colloidal stable oil-in-water (o/w) nanoemulsion system stabilized by two environmentally friendly ionic surfactants, i.e., amphoteric semi-synthetic coco-betaine (CB) and biotechnologically obtained anionic surfactin (S) (Scheme 1).

### 2.1. Supercritical Fluid Extraction—Obtaining the Purest Extracts

Analytical-scale SFE chosen for extraction from *P. corylifolia* seeds to obtain the purest possible bakuchiol-rich extract is a good alternative to conventional solvent-based extraction techniques [19,22]. The main advantages consist of higher mass transfer rates, increased selectivity, and minimal organic solvent consumption. Therefore, this green method of separation has gained remarkable interest among the scientific community in recent years. Another benefit of SFE lies in the use of CO_2_, in which most poorly water-soluble compounds dissolve. Most of these agents are not sufficiently stable at or near the critical temperature, and as a result, experimental measurements of their critical properties are extremely difficult. Thus, prediction methods must be developed to give reasonably accurate predictions for estimating the critical temperature, pressure, and volume of key components. The predicted solubility (in mole fraction) of bakuchiol and psoralen was in the range 10^−6^–10^−4^ for pressures of 220 to 300 bar, according to experimental data. Therefore, better solubility of bakuchiol in SC-CO_2_ at 300 bar was observed, though it was related to a significant increase in psoralen solubility [23].

Experiments carried out by other research groups indicate that SFE at temperatures of 45 °C and at 100 bar has given extracts containing bakuchiol as the main component, but with a 1.5% yield of extraction, whereas extraction and purification of psoralen and isopsoralen from *P. corylifolia* seeds required higher pressures (260–340 bar) and temperatures (up to 60 °C) [19]. After a careful analysis of the available data on supercritical extraction of *P. corylifolia* seeds, we decided to apply the following extraction conditions: 280 bar, 40 °C. In order to obtain the extract rich in bakuchiol and poor in psoralens, the static–dynamic procedure was applied.

The optimized parameters of SC-CO_2_ extraction are summarized in Table 1, and the chromatograms are shown in Figure S1 (see Supplementary Materials). Static extraction times were in the range of 10 to 15 min, followed by dynamic extraction times of 15 to 50 min. Comparable amounts of extracts were obtained at different static/dynamic cycles, but the highest yield (8.58%) was observed for sample 4 (10/20 intervals and 330 min of extraction at 280 bar). Changing the interval from 10/20 to 15/15 mode extended the extraction time with no significant yield increase, and lowering pressure caused decreasing yield and increased extraction extension. Obtained SC-CO_2_ extracts are oily dark red liquids with a content of approximately 80% bakuchiol and 5–6% psoralen and isopsoralen (analysis by HPLC in Supplementary Materials). A quite interesting observation occurred during the process: after decompression, a white precipitate was obtained in the extracts; HPLC-UV analysis confirmed the presence of almost 84% psoralen and 7% isopsoralen. Manohar’s group [19] performed single step SC-CO_2_ at 40 °C and 220, 260, 300 bar. Extractions resulted in 9.7, 11.5, and 11.8% yield of psoralens respectively, which is almost twice as high as those obtained in our experiment. However, when comparing the time of extractions: 1400, 855, 1085 min, respectively, it was 2–4 times longer. What is more, even such process time extensions did not cause the bakuchiol content to increase in the sample. Total bakuchiol content was 49.0, 41.1, and 51.7%, respectively, whereas in our experiments, it was above 80% in each sample. Manohar, in his study, had controlled bakuchiol content in the sample during the SC-CO_2_ extraction. Results showed that bakuchiol is extracted at the beginning of the process (above 60% of bakuchiol in each sample after 365, 200, 120 min). This shows that to obtain an extract with a high bakuchiol content, it is not necessary to obtain a high extraction yield. Our results are in line with this statement. Optimized extraction conditions resulted in obtaining extracts characterized by a high content of bakuchiol.

### 2.2. Physicochemical Characteristics and Optimization Studies

**Figure 1:**
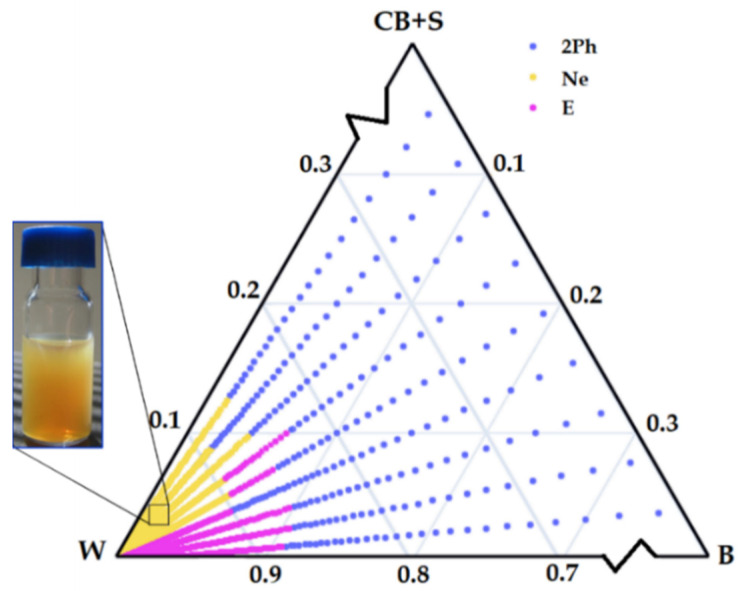
Partial phase diagram of the studied nanoemulsion stabilized with coco-betaine (CB) and surfactin (S) mixture (4:1), oleic phase with bakuchiol-rich extract (B), and water (W). Ne: oil-in-water type nanoemulsion (o/w), E: emulsion, 2Ph: double phases.

The effective delivery of the solubilized bakuchiol to skin target tissues depends on o/w formulation physicochemical properties such as particle shape, size distribution, surface charge, and colloidal stability, which were firmly established. The second step of our studies involved preparing a phase diagram with the water/oil/surfactant composition, using the aqueous titration method as a result of phase transitions produced during the spontaneous self-assembly process. Slow titration with aqueous phase was done for each weight ratio of oil (B) phase and surfactant (CB + S) mixture, and visual observations were carried out for semi-transparent, opalescent and bluish oil-in-water (o/w) nanoemulsion [13]. The physical state of the nanoemulsion (Ne) was marked on a pseudo-three-component phase diagram, with one axis representing the aqueous phase (W), the other showing oil (B), and the third representing the surfactant mixture (CB + S). The partial phase diagram of the nanosystem is shown in Figure 1.

Then, five different formulations from the o/w nanoemulsion region (Table 2) were chosen to provide the next step of our studies, i.e., dispersion stability tests according to the experimental procedure. The stability of the selected pseudoternary systems was observed for 72 h during six cycles between the refrigerator (4 °C) and room temperature (25 °C). Subsequently, those nanoemulsions that did not show any phase separations were centrifuged at 4000 rpm for 30 min. Finally, three of the most stable systems which showed no phase separation, creaming, coalescence, or phase inversion upon these tests were subsequently characterized with concern to the size of the droplets (D_H_), polydispersity (PdI), and ζ- experimentations. The compositions and physicochemical properties of these formulations are summarized in Table 2. Finally, the formulation with the lowest nanodroplet dispersion and highest zeta potential (system 4) was selected for further investigation. The small droplet size (200–300 nm), high surface charge, and near monodisperse size distribution of the obtained o/w nanoemulsion droplets make them good candidates for transdermal application [1,26].

**Figure 2:**
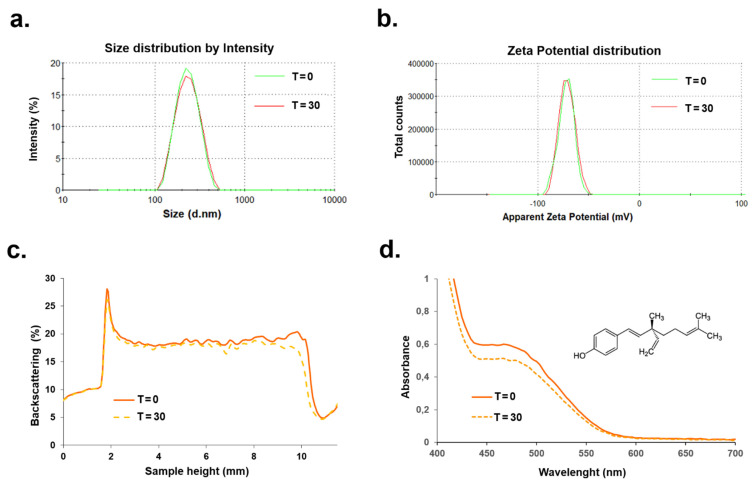
Characteristics of the optimized nanoemulsion system (sample 4) along with hydrodynamic diameter (D_H_) (**a**), zeta potential (**b**), backscattering profiles (**c**), and UV–Vis spectrum (**d**), analyzed for freshly prepared formulations (T = 0), and those stored for 30 days.

The physical stability of any nanocarrier system is extremely important for providing the maximum, long-lasting effectiveness of the active payload in the given transdermal application [26]. Consequently, the optimized nanoemulsion was then subjected to stability tests over 30 days involving scattering (DLS, ELS, BS) methods. The influence of storage time on mean particle size and ζ-potential for the optimized nanoemulsion was provided by DLS and ELS methods. As shown in Figure 2a,b, the obtained diameter (221 nm) and the zeta potential values (−73 mV) exhibited minimal changes over time. Additional studies on nanoemulsion flocculation, coalescence, sedimentation, or creaming processes were provided using a turbidimetric method, since these adverse phenomena that occur during storage time may affect the kinetic stability of the formulation [2,3].

The BS profiles of the optimized nanoemulsion were determined for a freshly prepared formulation, and the same system after its storage at room temperature. The representative Turbiscan plots are shown in Figure 2c as the levels of backscattering (expressed in %) marked on the ordinate axis and the nanoemulsion vial level (expressed in mm) indicated on the abscissa axis. From the plots, we do not observe a big separation between the curves, which indicates no evident particle growth or migration in nanoparticle dispersion, and thus, high kinetic stability [13]. This kinetic stability of the optimized formulation was confirmed by a low Turbiscan Stability Index (TSI), which was 1.2. As previously described, the lower the TSI, the more stable the formulation is obtained [27]. UV–Vis spectroscopy was used to demonstrate bakuchiol-rich extract loading in the nanoemulsion. Figure 2d presents the UV–Vis spectrum of the solubilized compound in the freshly prepared formulation compared with the spectrum of the same sample after 30-day storage. The representative peak at 480 nm was almost unchanged after the storage period, which provided evidence of the effective solubilization process of the hydrophobic cargo, as well as the preservative influence of the applied nanoemulsion formulation.

**Figure 3:**
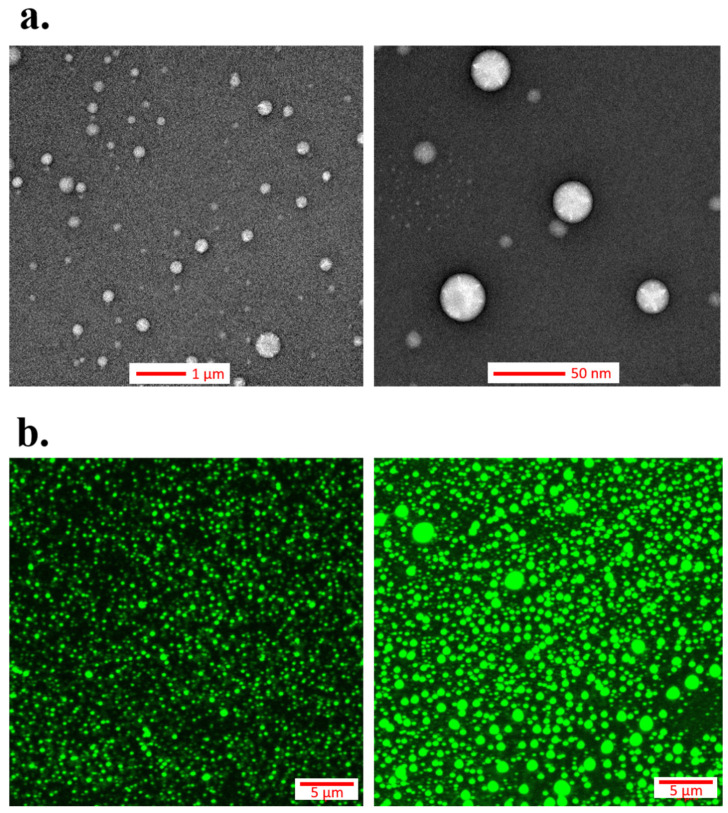
Transmission electron microscopy (TEM) (**a**) and confocal laser scanning microscopy (CLSM) (**b**) images of the optimized nanoemulsion system (sample 4).

The shape and morphology of the dispersed bakuchiol-rich extract droplets were imaged using transmission electron microscopy (TEM), as the most common method for evaluating any colloidal nanomaterial structure. The TEM images (Figure 3a) revealed spherical nanostructures with roughly uniform sizes and good droplet distribution. Additional fluorescent imaging performed by confocal microscopy—CLSM (Figure 3b)— confirmed the round shape of the nanodroplets. Furthermore, no punctuated green signals of aggregated nanodroplets were detected. Consequently, any coalescence or flocculation processes were not observed on the TEM and CLSM images, being in good agreement with BS evaluation. Moreover, the imaging data confirmed the results obtained by DLS and showed nanodroplets with sizes of approximately 200 nm. It is significant to take into account that a comparison of the results obtained in the wet (DLS) and dry conditions (TEM and CLSM) sometimes leads to an underestimation of the nanoemulsion diameter [12,13]. The obtained results showed that our optimized “green” formulation with bakuchiol is highly stable, and consequently, it was taken for the next step in our contribution, involving in vitro, ex vivo, and in vivo biological response studies upon skin cells and tissues. Furthermore, a nanoemulsion with analogous composition and stability (see Table S1 in Supplementary Materials), but containing retinol (as the commercially available active form of vitamin A) instead of bakuchiol, was used as a control for the biological experiments.

### 2.3. Evaluation of Skin Biological Responses: In Vitro, Ex Vivo, and In Vivo

The efficiency of the obtained nanoemulsion formulation in potential skin applications was tested through topical experiments involving biological skin responses In vitro, ex vivo, and in vivo. The first stage of the research was ex vivo tests of the prepared nanoemulsion penetration, which were carried out in a Franz chamber. The formulations tested were placed in the donor chambers. After 1 h and 7 h, the solutions were collected from the acceptor chamber and were subjected to HPLC analysis (for surfactin and coco-betaine) to confirm that the surfactants did not enter the bloodstream. The tested compounds were not identified in acceptor fluids. During the analysis, the formulations remained on the skin. In order to determine the location of the formulations in the skin after the performed permeability test in Franz cells, the skin was visualized by CLSM.

**Figure 4:**
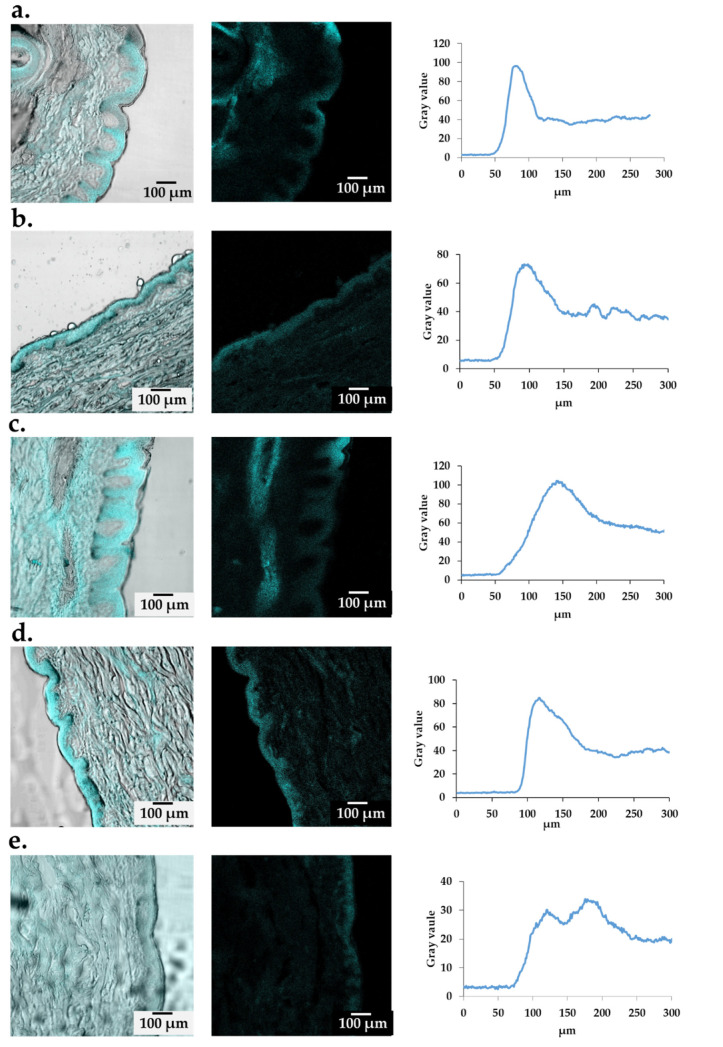
Penetration of nanoemulsion stained by coumarin—a fluorescent dye—in formulation with bakuchiol (**a**) after 1 h, (**b**) after 7 h, and with retinol (**c**) after 1 and (**d**) after 7 h, compared to the control (**e**).

Coumarin was added to both nanoemulsions to determine the location of the carriers in the skin after the permeability tests in the Franz chamber. The presence of fluorescent dye in skin tissues indicates that the prepared formulations can penetrate the epidermal barrier to the deeper layers of the skin. After 1 h following the application of the nanoemulsion with bakuchiol, we could see a continuous strip, located mainly in the SC (Figure 4a). After another 6 h, the fluorescence of the dye used became brighter—the fluorescence visibly situated in the deeper layers of the tissue (Figure 4b). In the case of nanoemulsions with retinol, scattered fluorescence could already be seen after 1 h, suggesting that the formulation with retinol had disintegrated, releasing the active ingredient (in this case a fluorescent dye) into the skin (Figure 4c). The retinol-loaded nanoemulsion did not reach the dermis layer in its original state (Figure 4d). In skin samples treated with a nanoformulation containing bakuchiol-rich extract, an increase in fluorescence could be observed. One hour after application, this increase was recorded at a depth of 80 microns (Figure 4a). We observe the carrier’s penetration into the skin at a depth of 80–120 µm. After 7 h, the intense fluorescence signal extends to a depth of approximately 140 µm, and the fluorescence intensity drops to approximately 70 (mean gray value) (Figure 4b). This decrease may indicate that the carrier is spread over a greater depth of the skin. In the case of skin treated with a carrier containing retinol, an increase in fluorescence can already be noticed 1 h after application, but at a greater depth of 80–180 µm than in the nanoformulation with bakuchiol-rich extract (Figure 4c). As mentioned before, this carrier disintegrated, releasing retinol. The maximum fluorescence value was slightly higher than in the nanoformulation with the extract containing bakuchiol. After another six hours, the fluorescence intensity decreased. However, the nanoformulation with bakuchiol was higher (above 80 mean gray value; Figure 4d). Fluorescence analysis of a control sample indicated skin autofluorescence. An increase in the signal on the border of the SC is observed (Figure 4e). The signal slightly increases to a maximum value of about 35 in the deeper layers, between 100 and 200 µm. Based on this analysis, it can be concluded that both carriers penetrated the SC. In the case of nanoemulsions with retinol, based on the fluorescence, it can be said that it penetrated the deeper layers of the skin faster, but it was probably caused by the rapid disintegration of the carrier, which may cause unfavorable penetration of this active substance into subsequent tissues. The loaded bakuchiol also effectively penetrated the skin, but it was more stable and allowed for the transport of the active substance in the intact carrier.

**Figure 5:**
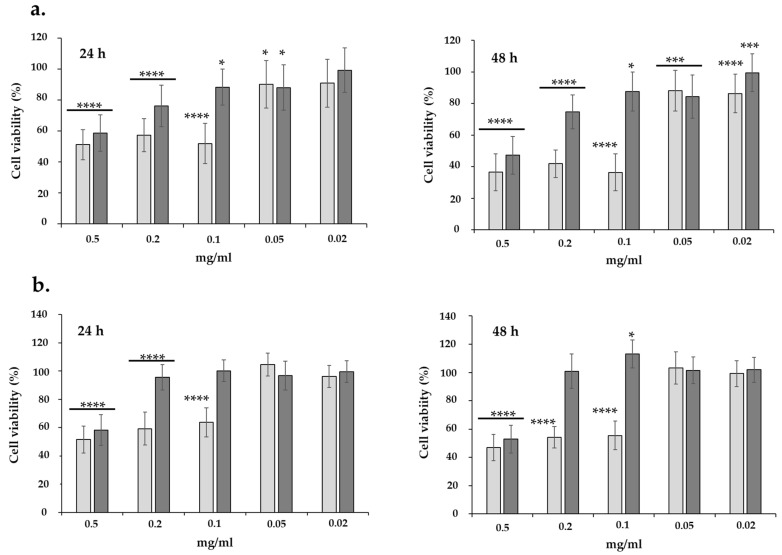
Cytotoxicity of the nanoemulsion with bakuchiol (dark grey) and control formulation with retinol (light grey) upon normal human skin cells: HaCaT keratinocytes (**a**) and NHDF fibroblasts (**b**), incubated for 24 h and 48 h. **** *p* < 0.0001, *** *p* < 0.0001, * *p* < 0.05.

The next step involved the cytotoxicity experiments, where nanoemulsions with different active compounds, i.e., bakuchiol-rich or retinol (applied as a control) were tested after 24 h and 48 h in cell cultures. The formulation concentrations were between 0.02–0.5 mg/mL during testing. The results presented in Figure 5 indicate the effects of the loaded formulation on the viability of HaCaT and NHDF cell lines. The MTT assay revealed relatively low cytotoxicity of the encapsulated bakuchiol even at the highest concentration (0.5 mg/mL) against both cell lines compared to the formulation containing retinol. The viability of the cells decreased to ca. 60% (after 24 h) and 55% (after 48 h) in comparison to untreated cells. At a lower concentration (0.02–0.2 mg/mL), we observed excellent cell survival and even a beneficial effect of the tested formulation on their growth, exceeding 100% of the control in the case of NHDF cells, at both times tested. The HaCaT and NHDF cells showed much lower tolerance concerning retinol—a big decrease in cell viability was noted for formulations from 0.5 to 0.01 mg/mL, after 24 and 48 h. Only at concentrations starting from 0.05 mg/mL can we speak of a comparable tolerance of cells to the tested formulations with bakuchiol and retinol. Generally, the cytotoxicity of the retinol formulation was slightly higher for HaCaT (Figure 5a), than the NHDF cell line (Figure 5b), especially after 48 h of cell treatment. These results seem to confirm the experiments described above for the skin permeability test on Franz cells and visualization of penetration of nanoemulsion into the skin. These studies showed rapid retinol release from the formulation, which may cause higher skin irritation than bakuchiol formulations. Thus, we can state that the applied nanoemulsion with bakuchiol was more biocompatible for both normal cell types used in the study.

Retinol is commercially produced and administered as esters, such as retinyl acetate or palmitate and retinoic acid. It should be noted that both vitamin A/retinol derivatives have been suggested to be irritating, skin-affecting, and cancer-preventative agents, especially when combined with sunlight [28]. Consequently, the application of a novel plant-origin alternative with similar medical and cosmeceutical properties is badly needed. In our study, the retinol-loaded nanoemulsion was used to compare obtained results with literature data. Additionally, researchers recently indicated that retinyl palmitate used in a nanoemulsion didn’t affect a 3T3 mouse fibroblast cell line nor a HaCaT cell line at concentrations below 2.5 mg/mL after 24 h of treatment [29]. Another nanoemulsion containing vitamin A as an active ingredient showed that the viability of MAC-T (bovine mammary epithelial) cells significantly decreased at concentrations of 200–500 µg/mL [30]. On the other hand, bakuchiol was found to have no cytotoxic activity against B16 melanoma cells at up to 3–10 mg/mL (IC_50_ 5.9 mg/mL) [31]. Babchi oil, a source of bakuchiol, was tested against the HaCaT cell line and showed cytotoxicity with IC_50_ at 172.3 µg/mL. After encapsulating babchi oil into nanosponges, cytotoxicity decreased slightly; the IC_50_ value was 191.4 µg/mL [32]. These results did not show a significant difference between the cytotoxicity of Babchi oil alone and a nanoformulation with this oil, which indicates the release of a sufficient amount of active ingredient (in this case Babchi oil), which caused cytotoxicity. Based on these tests, the authors concluded that the developed nanoformulation is safer for human skin cells than the oil alone.

**Figure 6:**
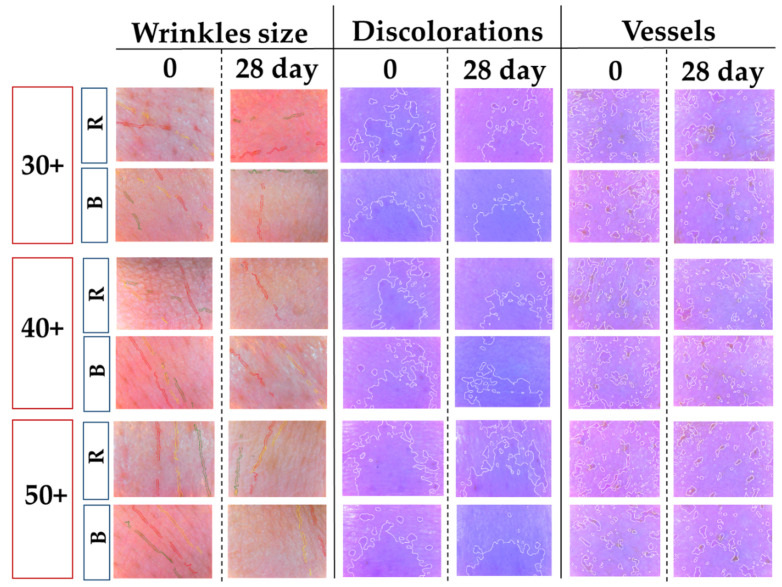
Selected images presenting changes in skin parameters after using nanoemulsions with retinol (R) and bakuchiol (B).

The third phase of our investigation involved an in vivo skin interaction study (Figure 6, Table 3). As previously shown, nanoemulsions can penetrate the epidermis barrier and deliver active ingredients deep into the skin [12]. In the in vivo study, the effect of nanoemulsion loaded by retinol or bakuchiol was evaluated. Their effects on wrinkles, capillaries, and skin discoloration were tested. At the outset, it should be noted that the effectiveness of a given preparation depends, inter alia, on the condition of the skin before application, age, size of the changes, etc. People around the age of 30 usually notice the first slight signs of skin deterioration, e.g., wrinkles. However, these changes are often minor, so a smaller effect of a given preparation can be observed. On the skin of people over 40, changes in skin condition are usually visible to the naked eye, so that such people may notice larger changes in their skin during longer use of the preparation. The skin of people over 50 most often already has quite permanent changes, hence the effect of the preparation may be observed as smaller compared to younger respondents [33].

As shown in Figure 6 and Table 3, both the formulations developed in our study improved the condition of the skin. The use of nanoemulsions with retinol influenced the depth of wrinkles, reducing them in all subjects at their 30 s, 40 s, and 50 s years old. The blood vessels in the skin also decreased. The preparation with bakuchiol also had a positive effect on wrinkles and blood vessels in the skin of the respondents. However, in the case of discoloration, a reduction was observed in a 30-year-old person, whereas the changes regressed when using the preparation with retinol. The performed experiments suggest that the nanoemulsion with bakuchiol is a promising formulation applicable in cosmetics. The study, however, is an introduction, and further research on people of a similar age is needed to finally verify the preliminary results.

## 3. Materials and Methods

### 3.1. Chemicals

Coco-betaine (alkyldimethyl betaine) applied as an amphoteric surfactant was a kind gift from PCC Exol Rokita (Poland). Surfactin used as an anionic biosurfactant was obtained in a biotechnological manner from *Bacillus subtilis* natto KB1 strains grown on rapeseed meal, according to the procedure described below. The bakuchiol-rich extract obtained via supercritical fluid extraction (the detailed procedure was indicated below) was used as the oil active phase. Raw Babchi seeds (*Psoralea corylifolia*, L.) obtained from NANGA herbal wholesaler (Złotów, Poland), were ground to obtain a particle size < 1 mm (Ultra Centrifugal Mill ZM 200, Retsch GmbH, Haan, Germany). Grinded samples were stored at −20 °C until further use. Carbon dioxide (98% purity), purchased from ZT Propan (Bydgoszcz, Poland) was employed for the supercritical fluid extraction. Supplemental chemical compounds were of commercial grade and were used as received. Water used for all experiments was doubly distilled and purified by means of a Millipore (Bedford, MA) Milli-Q purification system.

### 3.2. Surfactin Synthesis

#### 3.2.1. Synthetic Protocol

Surfactin (S) was obtained from *Bacillus subtilis* natto KB1 strains grown on rapeseed meal. Bacteria cultivation was carried out in modified Landy’s medium [11]. The preculture was carried out overnight at 37 °C with shaking at 180 rpm in LB (Luria-Bertani) medium (10 g/L bacto-tryptone, 5 g/L bacto-yeast extract, 10 g/L NaCl). The starting optical density in Landy’s medium was set to OD_600 nm_ = 0.1. Cultures were grown in 1 L baffled conical flasks with 250 mL of bacterial cultures and run at 37 °C with continuous shaking at 200 rpm for 72 h. After this time, the cultures were centrifuged (14,000× *g*) at 4 °C for 30 min (Sigma 6K15, rotor 12500, DJB Labcare Ltd., Germany). The resulting supernatant was acidified to pH 2.0 and left for 24 h at 4 °C. The precipitate was centrifuged, 10 mL of MiliQ water was added, and then the acidic pH was neutralized with NaOH. It was then extracted 5 times with 200 mL ethyl acetate and the organic fractions were collected and evaporated. The residue pellets were dissolved with ultra-pure water and freeze-dried.

#### 3.2.2. Analytical Evaluation

High-performance liquid chromatography (HPLC) was used to identify and confirm the presence of surfactin. The system consisted of a Beckmann System Gold 126 pump module equipped with a Knauer variable wavelength monitor and a controller computer with LP-chrom software (Version 1.49, Lipopharm.pl, Gdańsk, Poland, 2017). The separation was performed on a Macherey–Nagel Nucleodur C18 Isis column (50 × 4.6 mm, 1.8 microns; Fisher Scientific, Göteborg, Sweden). An isocratic separation was applied without temperature control, using acetonitrile and 0.1% aqueous acetic acid as the mobile phase, flowing at a rate of 0.7 mL/min. The solvent ratio was 85:25 for MeCN /aqueous acetic acid. A UV–Vis detector at 205 nm was used for surfactin detection. The analysis of the obtained chromatograms was performed using LP-chrom software. The surfactin was analyzed and its concentration was calculated using the sample’s area under the curve (AUC; see Figure S2 in Supplementary Materials).

### 3.3. Supercritical CO_2_ Extraction Procedure (Bakuchiol-Rich Extract Fabrication)

#### 3.3.1. SC-CO_2_ Protocol

The process was performed in a Speed Helix™ supercritical fluid extractor (Applied Separations, Allentown, PA, USA) using a 300 mL cylinder extraction vessel and 80 g of powdered *Psoralea corylifolia* seeds for each extraction. The extraction protocol involved extraction with supercritical CO_2_ (pure). The SC-CO_2_ extraction conditions employed for these processes were 280 bar, 40 °C, with a CO_2_ flow rate of 4 L NPT/min (equivalent to 3.6 g/min). The extraction was conducted in static/dynamic cycles: for a duration of 10 or 15 min in static mode, followed by 15, 20, or 50 min in dynamic mode. During the dynamic interval, CO_2_ carrying the extract flowed out of the unit and into a pre-weighed 60 mL collection flask. A balance (precision of ± 0.0001 g) was used to weigh the extract at regular time intervals until weight gain was no longer observed. Extracts were stored in the dark at −20 °C.

#### 3.3.2. Analytical Evaluation

High-performance liquid chromatography (HPLC) was used to identify and confirm the presence of bakuchiol. The apparatus consisted of a Beckmann System Gold 126 pump module with a Knauer variable wavelength monitor and a control computer with LP-chrom software (version 1.49, Lipopharm.pl, Gdansk, Poland, 2017). The separation was performed on a Phenomenex Kinetex^®^ EVO C18 100 Å (150 × 4.6 mm, 5 microns) column (Aschaffenburg, Germany). A gradient flow was used; gradient elution program: 30–90% MeOH *v*/*v* in 10 min, 10–15 min-90% MeOH *v*/*v* 15–25 min: 90–30% MeOH *v*/*v*; 25–30 min-30% MeOH *v*/*v*. A UV–Vis detector at 261 nm was used for surfactin detection. The analysis of the obtained chromatograms was performed using LP-chrom software. The bakuchiol was analyzed and its concentration was calculated using the sample’s area under the curve (AUC; see Figure S1 in Supplementary Materials).

### 3.4. Ternary Phase Diagrams and Nanoemulsion Preparation

The phase behavior (ternary phase diagrams) of surfactant–oil–water (SOW) systems, containing different ratios of coco-betaine (CB) and surfactin (S) (4:1) as the surfactant (CB + S) mixture, bakuchiol (B)-rich extract as the oil phase, and a water (W) phase, were examined using the aqueous titration method described in our previous work [13]. Generally, the phase boundaries were determined by the stepwise addition of water to the weighted mixture of the other components. After each portion, the samples were stirred and kept in thermostatic baths at 25° to equilibrate. The amount of W phase added was varied to produce a water concentration in the range of 5–95% of total volume at around 5% intervals. The boundary lines drawn on the phase diagram lie equidistant between consecutive experimental measurements on either side of the phase boundary. After each 5% addition of the aqueous phase to the B:CB + S mixture and its equilibration, the visual observation was made. Through visual evaluation, the following categories were assigned: (1) Transparent and translucent with a blue shining appearance: oil-in-water (o/w) nanoemulsion (Ne); (2) milky or cloudy: emulsion (E); (3) two separate phases (2Ph).

### 3.5. Centrifugation Test and Heating and Cooling Cycles

To overcome the problem of metastable and unstable nanoemulsions, the selected five formulations (Table 2) were subjected to three dispersion stability evaluations: centrifugation, heating and cooling cycles, and freeze–thaw cycle tests [13]. The most stable compositions which showed no phase separation, creaming, coalescence, or phase inversion upon these tests were selected for further physicochemical analysis.

### 3.6. Nanoemulsion Characterization Methods

The physicochemical characteristics of the designed nanoemulsions involved scattering methods such as DLS (dynamic light scattering), ELS (Electrophoretic light scattering)*,* BS (backscattering), microscopic imaging by TEM (transmission electron microscopy), and CLSM (confocal laser scanning microscopy), according to the procedures detailed below.

#### 3.6.1. Dynamic Light Scattering (DLS) and Electrophoretic Light Scattering (ELS)

The average particle size (D_H_) and polydispersity index (PdI) values of the droplets were determined using DLS analysis. Zeta potential (ζ) evaluation was conducted using ELS measurements. Both the DLS and ELS protocols were made using a ZetaSizer Nano ZS (Malvern Instruments, Malvern, UK) equipped with a detection angle of 173°, and with a He-Ne laser (632.8 nm), and an ALV 5000 multibit, multitap autocorrelator (Malvern Instruments, Malvern, UK). All the measurements were taken at 25 °C. Each value was calculated as the average of three subsequent instrument runs with at least 20 runs.

#### 3.6.2. Backscattering (BS)

The nanoemulsions’ long-term physical stability was evaluated by backscattering profiles based on a turbidimetric method using a TurbiScanLab Expert (Formulaction SA, France) optical instrument. The BS method was applied to detect any destabilization in the obtained formulation such as creaming, sedimentation, flocculation, or coalescence phenomena in a few days [13,27]. The analyzed dispersion was placed on a cylindrical glass measuring cell, where its stability was monitored by measuring the backscattering of pulsed near-infrared light (λ = 880 nm). Two synchronous optical sensors receive, respectively, light transmitted through the sample (0° from the incident radiation, transmission sensor), and light back-scattered by the sample (135° from the incident radiation, backscattering detector). The experiment involves scanning the sample every minute at room temperature while moving along the entire height of the cell. Backscattering profiles (BS) as a function of sample height were collected and analyzed using the instrument’s software program (Turbisoft version 1.21). The same measurements were performed for freshly prepared nanoemulsions and repeated on the same samples after 30 days of storage at 25 °C.

#### 3.6.3. Transmission Electron Microscopy (TEM)

TEM imaging was performed using a FEI Tecnai G2 XTWIN transmission electron microscope (FEI, Hillsboro, OR, USA). Nanoemulsion morphology was determined by counting the size of approximately 250 droplets from several TEM images obtained from different sites on the grid. Energy dispersed X-ray (EDX) spectra were recorded with a Thermo Scientific Ultra Dry detector (Thermo Fisher Scientific, Waltham, MA, USA) (resolution 129 eV) and analyzed with the Noran System 7 (Thermo Fisher Scientific, Waltham, MA, USA). The samples were prepared by placing a small amount of diluted nanoemulsion dispersions on a Cu–Ni grid and stained with 2% uranyl acetate before shooting. Size distribution plots were fitted using a Gaussian curve approximation.

#### 3.6.4. Confocal Laser Scanning Microscopy (CLSM)

CLSM was used for additional visualization of the nanoformulation and for ex vivo penetration studies. To visualize the nanoemulsions, coumarin was added as a fluorescent marker. The nanoemulsions were observed under a Leica SP8 LSM microscope (Leica microsystems) using a 405 nm excitation laser and an emission gate between 420 and 560 nm. To visualize the location of the carriers in skin tissue, saturated solution of coumarin in oil phase was added to the carriers containing both bakuchiol and retinol. The skin was observed under the Leica SP8 LSM microscope, using a 405 nm excitation laser and an emission gate between 420–560 nm.

### 3.7. In Vitro, Ex Vivo, and In Vivo Topical Experiments

The dermal biological response to the formulated nanoemulsion systems was evaluated during in vitro cytotoxicity experiments using human skin cell lines, i.e., HaCaT keratinocytes and normal human dermal fibroblasts NHDF, as well as via ex vivo permeation studies using transdermal Franz diffusion cells, and ex vivo penetration experiments by CLSM. Both the ex vivo experiments were performed on full-thickness pig skin. The in vivo interaction studies involved the evaluation of skin hydration, the size of wrinkles, and skin discoloration. The studies were accomplished involving human volunteers aged 30–50 years. A detailed description of the performed experiments is indicated below.

#### 3.7.1. Cell Cultures

In vitro studies were performed on a normal human dermal fibroblast (NHDF, Lonza, Warsaw, Poland) cell line and immortalized human keratinocytes (HaCaT, LGC Standards, Łomianki, Poland). The NHDF cells were cultured in minimum essential medium Eagle (EMEM) with alpha modifications, supplemented with 10% fetal bovine serum (FBS), glutamine (2 mM), penicillin (100 U/mL), and streptomycin (100 μg/mL). The HaCaT cell line was cultured in Dulbecco’s modified Eagle’s medium (DMEM) with 10% FBS, and glutamine (2 mM), penicillin (100 U/mL), and streptomycin (100 μg/mL). The cells were cultured in a 37 °C incubator in a humidified atmosphere containing 5% CO_2_. For the experiment, the skin in vitro cultures were seeded onto 96-well culture plates at 6 × 10^3^ and 4 × 10^3^ cells/well for NHDF and HaCaT cells, respectively, and incubated at 37 °C in a humidified atmosphere and 5% CO_2_ overnight.

#### 3.7.2. Colorimetric Cell Viability Assay

The in vitro cytotoxicity evaluation of the studied nanoemulsions was performed using a 3-[4,5-dimethylthiazol-2-yl]-2,5 diphenyl tetrazolium bromide (MTT) cell viability assay as in our previous studies [12,34]. For the experiments, human skin cells were removed by trypsinization (trypsin 0.025% and EDTA 0.002% solution) and rinsed twice with PBS. For cytotoxicity evaluation, both the cell types were seeded onto 96-well plates at a density described in 2.7.1., in 200 µL of medium/well and incubated at 37 °C in 5% CO_2_ atmosphere. Then, the cultured cells were treated with formulations loaded by a bakuchiol-rich extract or retinol to obtain a final dilution of 1:20, 1:50, 1:100, 1:200, and 1:500 (corresponding to the final 0.5–0.02 mg/mL concentrations of the active substances), and incubated for 24 and 48 h at 37 °C. Cell viability was determined by the MTT assay by measuring the absorbance of the resulting solutions at a wavelength of 570 nm using an ASYS UWM 340 microplate reader (Biogenet, Józefów, Poland). MTT solution (50 μL) was added to each well, which was then incubated at 37 °C for 4 h. After the incubation medium was removed, 50 µL of DMSO was added to dissolve formazan crystals. The untreated cells were normalized to 100% for each assay, and treatments were expressed as the percentage of the control. All experiments were in triplicate, and the data were expressed as mean values ± standard deviations.

#### 3.7.3. Ex Vivo Diffusion Test on Franz Cells

Experiments were performed on full-thickness pig skin. It was cleaned with tap water and then separated from the cartilage underneath. Then, the fat layer was separated and the skin was stored at −20 °C for up to 1 month. The prepared skins before testing were thawed to room temperature and were then hydrated with 0.05 M phosphate buffer (pH 7.4) for 30 min. The skin was trimmed to an appropriate size and secured between the donor and acceptor chambers in transdermal Franz diffusion cells (Hanson Research Corporation, Chatsworth, CA, USA). The system was equipped with a heating circuit with vertical glass diffusion cells with a jacket. A 0.05 M phosphate buffer at pH 7.4 was placed in the acceptor chamber and was gently stirred with a magnetic stirrer at 350 rpm. Then 0.5 mL of the formulation with nanoemulsion was introduced into the donor chamber. The temperature in the system was set at 32.5 °C to mimic the thermo-condition of the normal unclothed skin surface. The study was conducted for 1 and 7 h. The fluid taken from the Franz acceptor chamber was subjected to HPLC to identify the surfactin in the acceptor fluid (Figure S3 in Supplementary Materials).

#### 3.7.4. Ex Vivo Penetration Studies by Confocal Laser Scanning Microscopy (CLSM)

CLSM analysis was performed to visualize the penetration of the formulation (containing bakuchiol-rich extract and retinol) into the skin. The skin after ex vivo transdermal studies was protected by treatment with 4% (*w*/*v*) paraformaldehyde solution in PBS for 45 min at room temperature. The skin was then rinsed three times for 10 min with PBS (pH 7.4). The samples were then placed in 30% (*w*/*v*) sucrose in PBS and left overnight at 4 °C. The next day, the samples were embedded in a cryopreservation solution (Sakura, Torrance, CA, USA), placed in a cryomold, and frozen. The samples were cut into sections in a cryostat at −20 °C and placed on Superfrost Plus microscope slides (Sigma-Aldrich, St. Louis, MO, USA). The Leica SP8 LSM microscope (Leica microsystems) was used for imaging. The images were processed using ImageJ software. The fluorescence intensity was measured at three different sites, from which mean values were calculated. Fluorescence intensity was presented as a mean gray value.

#### 3.7.5. In Vivo Skin Contact Study

To investigate the effect of the formulation on human skin, in vivo tests were carried out using the NatiV3 Analyzer (Beauty of Science, Wrocław, Poland); this analyzer is a comprehensive device for computer cosmetology diagnostics. Volunteers aged 30–50 participated in the study. Based on in vitro tests, formulations with a concentration of active substances of 0.05 mg/mL were selected and the effects of both were compared with each other. 1 mL of the given formulation was applied with a glass pipette and rubbed over the skin. A 1 cm × 1 cm square matrix was used to determine the area of the skin covered with the preparation. The nanoemulsion was applied daily, morning and evening, for 28 days by both women and men. Skin analysis was performed before the start of the tests and on the 28th day of the test. Skin parameters, such as the size of wrinkles and skin discoloration, were assessed. The study protocol was approved by the Bioethics Commission at the Lower Silesian Chamber of Physicians and Dentists (1/PNHAB/2020).

### 3.8. Statistical Analysis

All the data are expressed as mean values ± standard deviation of three measurements. Statistical analyses were performed using one-way analysis of variance with post-hoc Tukey’s significant difference calculator (GraphPad Prism). A value of *p* < 0.05 was considered to be statistically significant.

## 4. Conclusions

The results presented in this contribution focused on the rational design and engineering of novel ecological and biocompatible oil-in-water nanoemulsions, consisting of sustainable materials gained from renewable sources, dedicated to solubilization and enhanced delivery of bakuchiol, the so-called “botanical” retinol, in topical applications. The “environmentally-friendly” nanoformulation was obtained by self-assembly of “green” hybrid ionic surfactants, i.e., coco-betaine obtained from coconut oil and surfactin molecules synthesized from rapeseed oil according to Green Chemistry principles. An important objective of this work was the bakuchiol synthesis using an ultra-modern supercritical CO_2_ extraction approach with pure carbon dioxide to show a “green” alternative to conventional solvent-based extraction techniques. As a result, we obtained a water-dispersible and kinetically stable bakuchiol formulation with a size of about 200 nm and a highly negative charge below 70 mV that offers: (i) excellent skin biocompatibility with the final 0.02–0.2 mg/mL active substance concentration, as indicated via in vitro tests upon human skin cell lines (HaCaT keratinocytes and NHDF fibroblasts), (ii) improved skin permeability as revealed in permeation studies, performed by ex vivo diffusion tests in Franz cells, and finally (iii) anti-aging, moisturizing, and skin-restoring ability, as demonstrated via in vivo interaction studies involving human volunteers. Hence, we strongly believe that this well-defined “green” composition can be valuable for comprehensive cosmetic applications and a sustainable alternative to conventional retinol products, with more ecological nature and less harmful effects such as skin irritation, photosensitivity, and health concerns.

## Figures and Tables

**Table 1:** Summary of SC-CO_2_ extraction with 40 ± 1 [°C] (extr vessel) and CO_2_ flow rate 2 ± 0.5 [L NPT/min] (dynamic extr.).

Sample Number/ Seed Weight (g)	P (bar)	Static/Dynamic Interval (min/min)	Extraction Time (min)	Extract (g)	Yield (%)	Bakuchiol (%)	Psoralen (%)	Iso-Psoralen (%)	Sum of the Other Components (%)
1/80.003	280 ± 5	10/50	250	4.731	5.91	79.68	1.23	3.14	15.95
2/80.003	280 ± 5	15/15	420	5.035	6.29	80.96	1.52	4.15	13.37
3/80.005	280 ± 5	10/20	330	6.862	8.58	80.25	1.23	3.15	15.37
4/80.002	250 ± 5	10/20	420	5.257	6.57	81.42	1.65	4.27	12.66

**Table 2:** Characteristics of the selected nanoemulsion samples.

System	Nanoemulsion Composition/%			D_H_^d^/nm	PdI ^e^	ζ ^f^/mV
	S ^a^	O ^b^	W ^c^			
1	10	5	85	^g^	-	-
2	10	2	88	-	-	-
3	5	2	93	243 ± 6	0.221 ± 0.02	−66 ± 3
4	5	1	94	221 ± 4	0.182 ± 0.01	−73 ± 5
5	3	1	96	200 ± 3	0.276 ± 0.02	−70 ± 5

^a^ Surfactant, ^b^ Oil, ^c^ Water, ^d^ D_H_: hydrodynamic diameter (Z-Ave), ^e^ PdI: polydispersity index, ^f^ ζ: zeta potential, ^g^ phase separation.

**Table 3:** Impact on wrinkles, discoloration, vessels.

Probant Age	Active Ingredient	Wrinkles (mm)		Discolorations (%)		Vessels (mm^2^)	
		0	28th Day	0	28th Day	0	28th Day
30+	R	0.085 ± 0.018	0.082 ± 0.02	10.87 ± 1.44	10.48 ± 1.36	2.61 ± 1.36	2.58 ± 1.19
	B	0.086 ± 0.013	0.082 ± 0.017	10.97 ± 1.65	10.28 ± 1.79	2.04 ± 1.18	1.90 ± 1.35
40+	R	0.100 ± 0.020	0.070 ± 0.040	2.87 ± 1.24	2.05 ± 1,63	11.51 ± 1.92	10.95 ± 1.31
	B	0.090 ± 0.010	0.090 ± 0.020	2.87 ± 1.24	2.05 ± 1.63	10.60 ± 1,21	10.85 ± 1.20
50+	R	0.099 ± 0.010	0.079 ± 0.035	12.23 ± 1.76	12.03 ± 1.57	2.82 ± 0.77	2.52 ± 1.35
	B	0.098 ± 0.013	0.092 ± 0.007	12.34 ± 1.9	10.75 ± 1.71	3.68 ± 1.39	3.44 ± 0.75

## Supplementary Materials

The following are available online at https://www.mdpi.com/article/10.3390/ijms221810091/s1.
