# Supplementary material for: Design and Engineering of “Green” Nanoemulsions for Enhanced Topical Delivery of Bakuchiol Achieved in a Sustainable Manner: A Novel Eco-Friendly Approach to Bioretinol
Source: Int J Mol Sci. 2021 Sep 18;22(18):10091. doi: 10.3390/ijms221810091 (PMC8472396; doi:10.3390/ijms221810091)
Supplement: Supplementary file 1 [file ijms-22-10091-s001.zip › ijms-1355036-supplementary.pdf]

Supplementary Materials

Design and Engineering of "Green" Nanoemulsions for Enhanced Topical Delivery of Bakuchiol Achieved in a Sustainable Manner: A Novel Eco-Friendly Approach to Bioretinol

Agnieszka Lewińska [1,*], Marta Domżał-Kędzia [2], Ewa Maciejczyk [3], Marcin Łukaszewicz [2] and Urszula Bazylińska[4,*]

[1] Faculty of Chemistry, University of Wroclaw, Joliot-Curie 14, 50-383 Wroclaw, Poland

[2] Department of Biotransformation, Faculty of Biotechnology, University of Wroclaw, Joliot-Curie 14a, 50-383 Wroclaw, Poland; marta.domzal@uwr.edu.pl (M.D.-K.); marcin.lukaszewicz@uwr.edu.pl (M.Ł.)

[3] Institute of Natural Products and Cosmetics, Faculty of Biotechnology and Food Sciences, Lodz University of Technology, Stefanowskiego 2/22, 90-924 Lodz, Poland; ewa.maciejczyk@p.lodz.pl

[4] Laboratory of Nanocolloids and Disperse Systems, Department of Physical and Quantum Chemistry, Faculty of Chemistry, Wroclaw University of Science and Technology, Wybrzeze Wyspianskiego 27, 50-370 Wroclaw, Poland

* Correspondence: agnieszka.lewinska@chem.uni.wroc.pl (A.L.); urszula.bazylinska@pwr.edu.pl (U.B.)

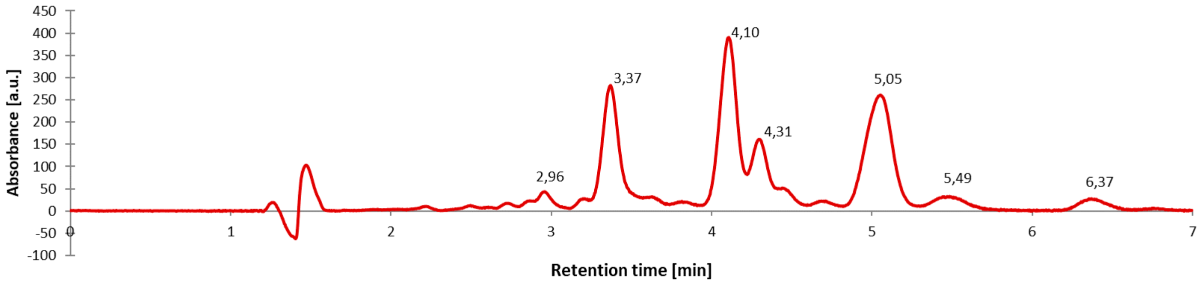

Fig. S1. Analytical chromatogram of the synthesized surfactin.

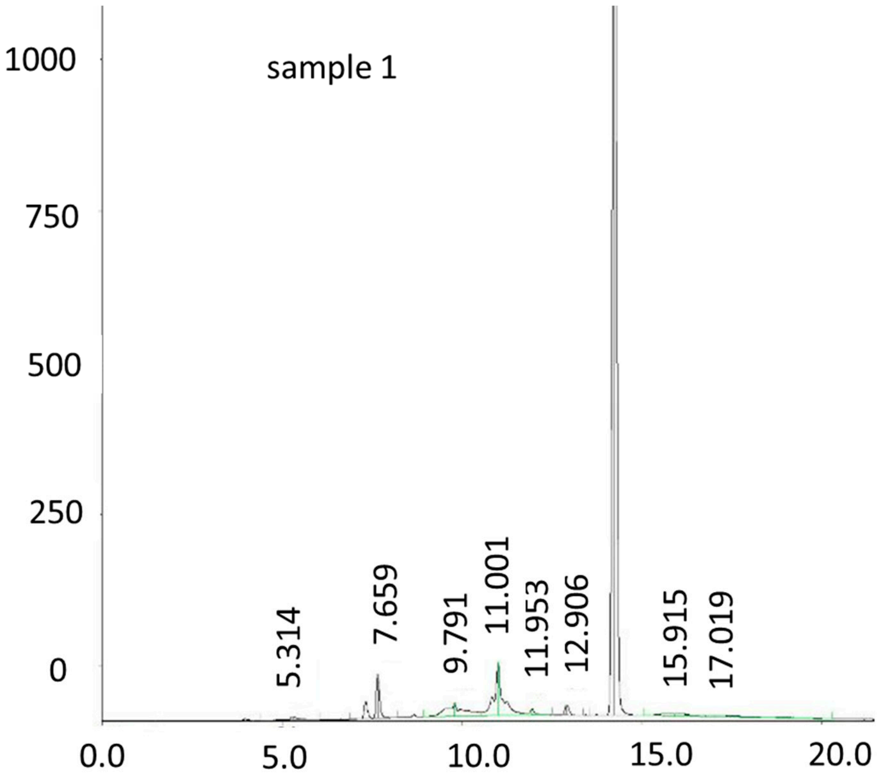
sample 1
1000
750
500
250
0
5.314
7.659
9.791
11.001
11.953
12.906
15.915
17.019
0.0
5.0
10.0
15.0
20.0

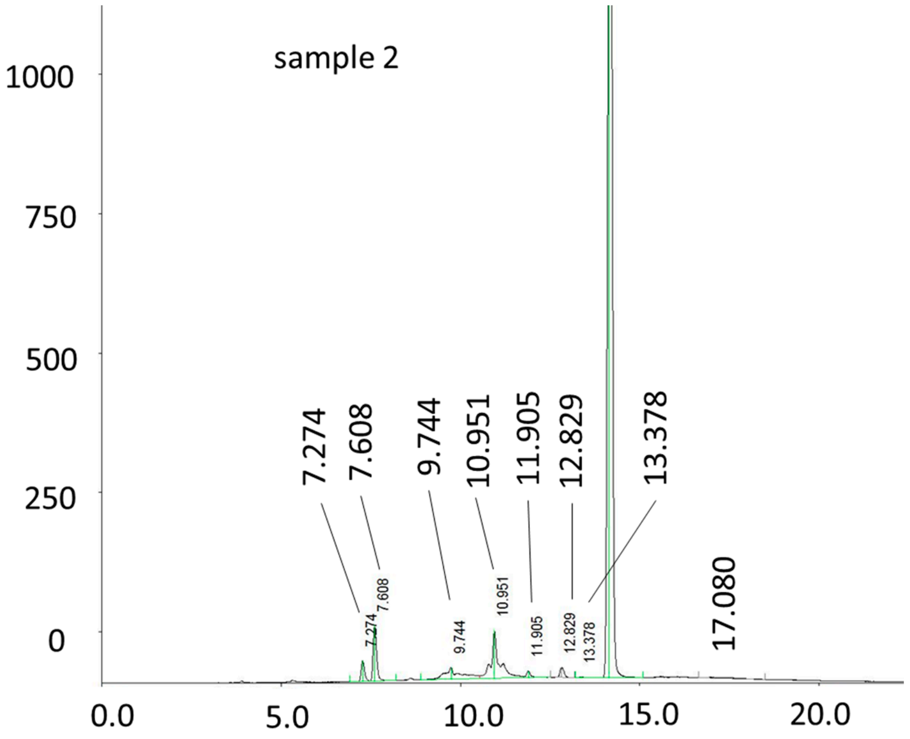
sample 2
1000
750
500
250
0
7.274
7.608
9.744
10.951
11.905
12.829
13.378
17.080
0.0
5.0
10.0
15.0
20.0

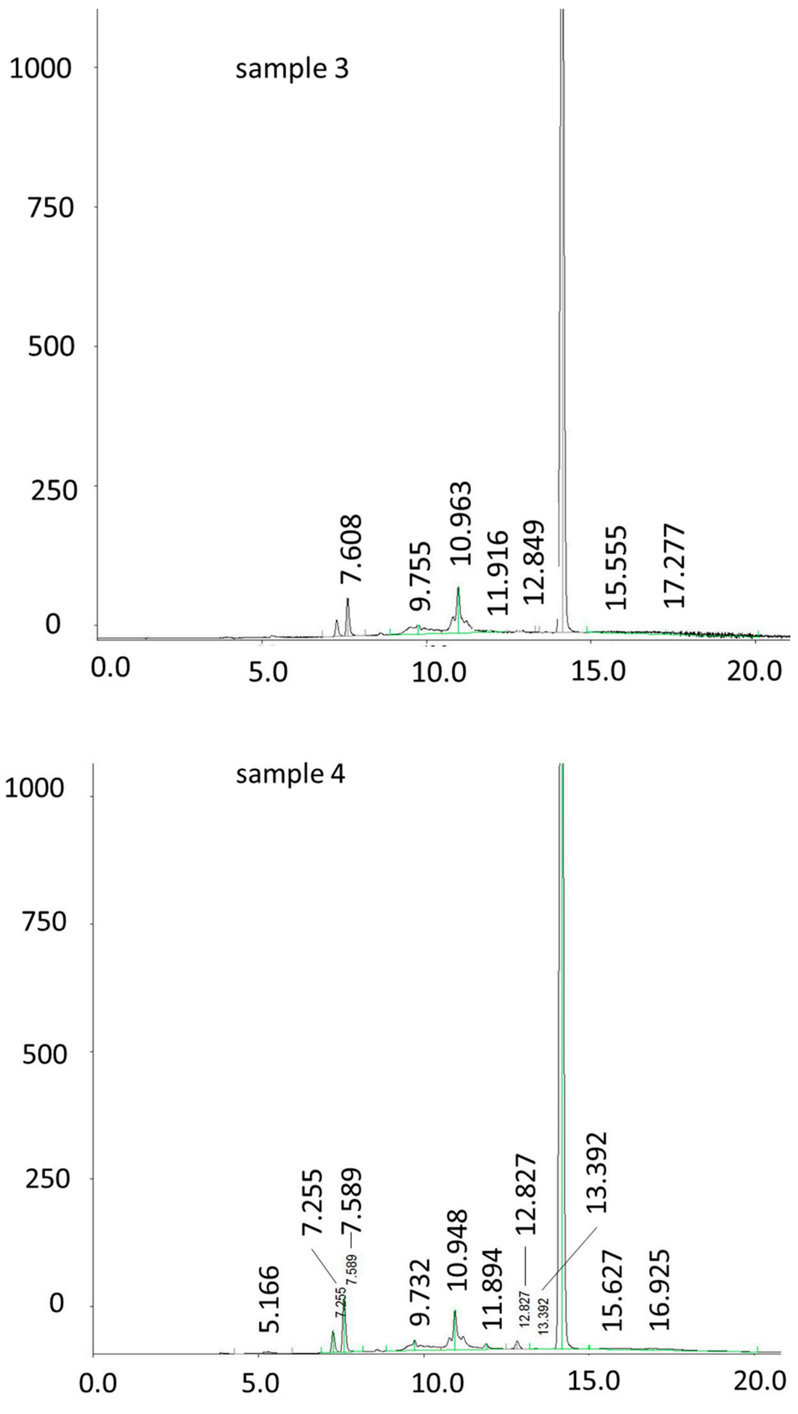

Fig. S2. Chromatogram of bakuchiol extracts from SC-CO_2 extraction.

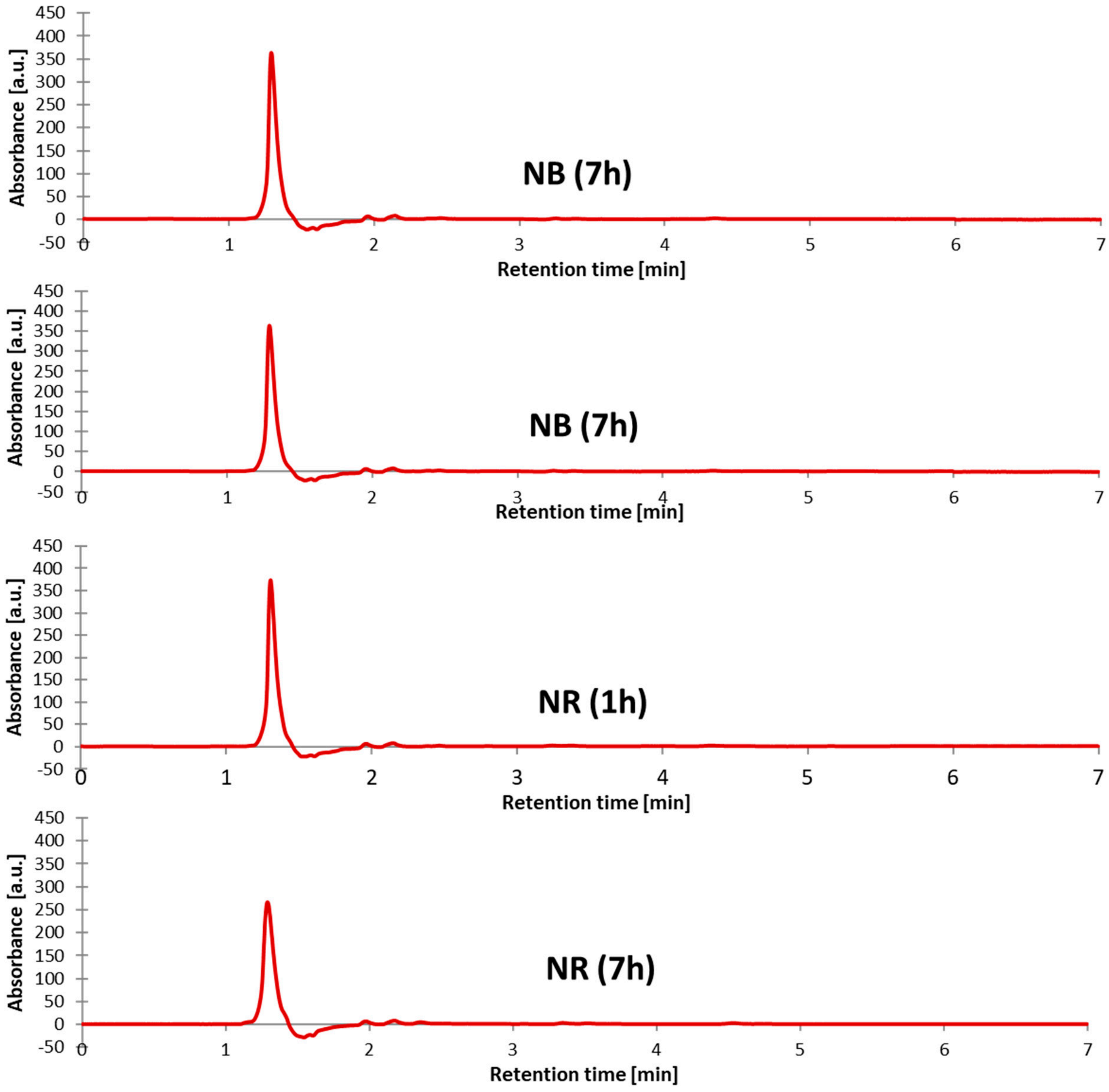

Fig. S3. Analytical chromatogram fluid from the Franz acceptor chamber - identify the surfactin in the acceptor fluid.

Table S1. Characteristics of the control nanoemulsion with retinol.

System	Nanoemulsion composition / %			D_H [d] / nm	PdI [e]	ζ [f] / mV
	S [a]	O [b]	W [c]			
**T= 0 days**						
4S	5	1	94	225	0,194	-72
**T= 30 days**						
4S	5	1	94	228	0,197	-77

[a] Surfactant, [b] Oil, [c] Water, [d] D_H: hydrodynamic diameter (Z-Ave). [e] PdI: polydispersity index. [f] ζ: zeta potential.
